# A real-time dataset of air quality index monitoring using IoT and machine learning in the perspective of Bangladesh

**DOI:** 10.1016/j.dib.2024.110578

**Published:** 2024-06-13

**Authors:** Md. Monirul Islam, Ferdaus Anam Jibon, M. Masud Tarek, Muntasir Hasan Kanchan, Shalah Uddin Perbhez Shakil

**Affiliations:** a Department of Software Engineering, Daffodil International University, Daffodil Smart City (DSC), Birulia, Savar, Dhaka 1216, Bangladesh; b Department of Computer Science and Engineering, IUBAT— International University of Business Agriculture and Technology, 4 Embankment Drive Road, Sector-10, Uttara Model Town, Dhaka 1230, Bangladesh; c Department of Computer Science and Engineering, State University of Bangladesh, South Purbachal, Kanchan, Dhaka 1461, Bangladesh; d Rising Research Lab, Sheikh Bari, Malancha, Melandah, Jamalpur- 2012, Mymensingh, Bangladesh

**Keywords:** Air quality index (AQI), Air pollution, Machine learning, IoT sensors

## Abstract

This paper produces a real-time air quality index dataset of three places named Kuril Bishow Road, Uttara, and Tongi in Dhaka and Gazipur City, Bangladesh. The IoT framework consists of MQ9, MQ135, MQ131, and dust or PM sensors with an Arduino microcontroller to collect real data on sulfur dioxide, carbon monoxide, nitrogen dioxide, ozone, particle matters 2.5 and 10 µm. The data is stored in an Excel file as a comma-separated file and after that, authors applied regression type and classification type machine learning algorithms to analyze the data. The dataset consists of 11 columns and 155,406 rows, where sulfur dioxide, carbon monoxide, nitrogen dioxide, ozone, and particle matter 2.5 and 10 are recorded where AQI is marked as the target variable and the others are indicated as independent variables. In the dataset, AQI is categorized into five classes named Good, satisfactory, Moderate, Poor and Very Poor. After experimental results, it is seen that two places including Uttara and Kuril are comparatively suitable for Air Quality among the three places as well as the Random Forest algorithm outperforms the models. The study describes details of the embedded system's hardware as well. This dataset will be beneficial for environmental researchers to use to analyze the air quality.

Specifications Table

**Table:** 

Subject	Applied Machine Learning, Air Quality Monitoring, Embedded Systems using IoT Devices.
Specific subject area	Smart Environment Monitoring, IoT devices for air quality, Machine Learning.
Type of data	Comma Separated Value (CSV)
Data collection	An IoT Framework; IoT devices are employed in the data collection process to obtain real-time data on air pollution in Dhaka and Gazipur district of Bangladesh. To monitor levels of carbon monoxide, nitrogen dioxide, ozone, particulate matter 2.5, and particulate matter 10, the hardware design is created to guarantee that appropriate sensors were installed. The Arduino IDE code is uploaded to allow for communication with IoT devices. The Excel data streamer accessed the requires Excel file. The Arduino IDE selects the appropriate COM port to connect the IoT devices to the PC. The data streamer toolbar's “Start Data” option enables real-time data streaming into the Excel sheet. The "Record Data" button launches the data-collecting process, allowing IoT devices to continuously monitor and record air pollutants. To end data gathering, the “Stop Recording” button is pressed. The "Stop Data" function is used to halt data transmission between IoT devices and computers. The collected data is saved in a specified file location for easy analysis and interpretation.
Data source location	Institution: Department of Software Engineering, Daffodil International University, Daffodil Smart City (DSC), Birulia, Savar, Dhaka 1216, Bangladesh.
Data accessibility	Repository name: **Mendeley Data** Data identification number: DOI: 10.17632/4r25×9sc7k.1 Direct URL to data: https://data.mendeley.com/datasets/4r25×9sc7k/1 [5]
Related research article	Shakil, S.U.P., Kashem, M.A., Islam, M.M., Nayan, N.M., Uddin, J., Investigation of Air Effluence Using IoT and Machine Learning. In: Miraz, M.H., Southall, G., Ali, M., Ware, A. (eds) Emerging Technologies in Computing. iCETiC 2023. Lecture Notes of the Institute for Computer Sciences, Social Informatics and Telecommunications Engineering, vol 538. Springer, Cham (2024): https://link.springer.com/chapter/10.1007/978–3–031–50,215–6_12

## 1 Value of the Data

• This dataset is useful for monitoring air quality, identifying pollution sources, and assessing the efficacy of pollution-control measures.
• These data can be used to establish air quality standards, create policies and regulations, and assess environmental compliance.
• The dataset is helpful for research students to analyze using machine learning (ML) or data science techniques to insight the data.
• This dataset will be used by researchers, scientists, and engineers for investigating the sources and consequences of air pollution, creating novel pollution control technology, and evaluating the efficacy of treatments.
• These data will be used by researchers to predict the air quality index (AQI) condition as well as the environment situations like good, very good, satisfied, bad, poor.

## 2 Background

The dataset's goal is to completely measure and monitor air quality in Bangladesh's Dhaka and Gazipur districts, with an emphasis on determining its acceptability for human habitation and overall environmental health. By measuring key air pollutants such as sulfur dioxide, carbon monoxide, nitrogen dioxide, ozone, and particulate matter (PM2.5 and PM10), the dataset aims to provide a thorough understanding of the Air Quality Index (AQI) and its variations across different districts. The dataset's analysis aims to evaluate the degree of compliance with existing regulatory norms and recommendations, as well as identify areas of concern and possible sources of pollution for targeted intervention and pollution management. The dataset seeks to provide important insights into the overall environmental quality of the examined locations by allowing real-time air quality data to be compared to standard values. It also seeks to encourage scientific research by assisting in the development of prediction models and analytical tools for projecting AQI values and tracking long-term trends in air quality. Finally, this dataset adds to larger initiatives to reduce air pollution and promote sustainable environmental practices in Bangladesh's cities and peri‑urban areas.

## 3 Data Description

The dataset consists of 11 columns and 155,406 rows, where sulfur dioxide, carbon monoxide, nitrogen dioxide, ozone, particle matters 2.5 and 10 are recorded as independent variables. This data is collected in every winter and summer season from January 2017 to December 2022. AQI indicates the dependent variable. The dataset is recorded in three places at Dhaka and Gazipur districts of Bangladesh. In the district of Dhaka, from Kuril Bishow Road and Uttara places, 103,611 features are recorded and in the district of Gazipur, from Tongi, 51,794 features are recorded.

**Fig. 1:**
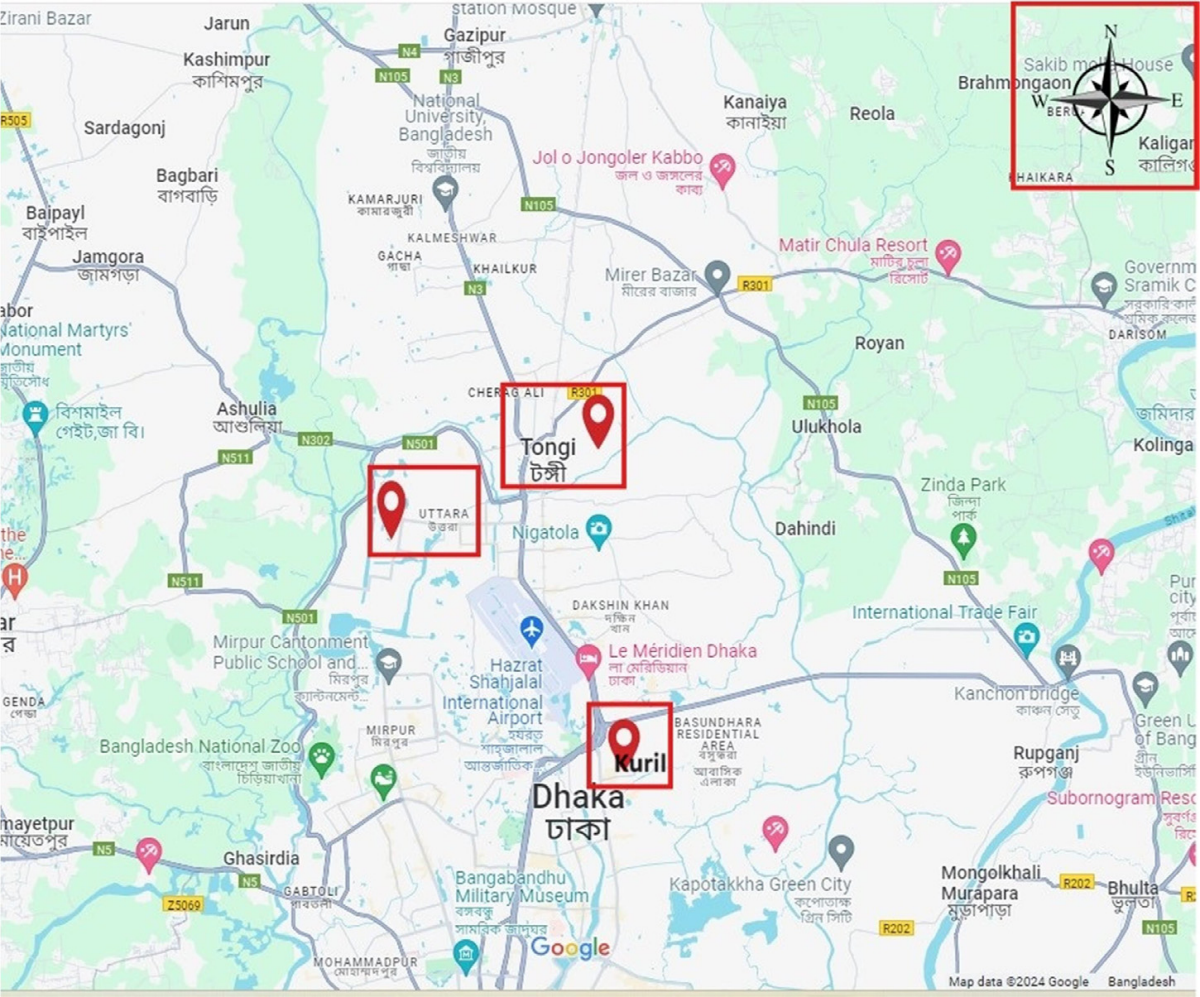
Location of data collection.

Using this dataset, a research paper is published [1]. The volume of the dataset can be increased with the help of paid cloud protocol as well as for a long time run of the system. Table 1 exhibits some portion of the dataset. The geographical area of collecting dataset is shown in Fig. 1. We denote the places by red colored rectangular. The top right corner rectangular denotes the direction of the map.

**Table 1:** Some data as sample.

SO2 (g/m3)	NO2 (g/m3)	CO (mg/m3)	O3 (g/m3)	PM2.5 (µm)	PM10 (µm)
0.04	0.056	3.9	0.0525	74	94
0.04	0.058	3.9	0.0525	74	93
0.04	0.058	1	0.0525	73	89
0.04	0.057	1	0.0525	72	91
0.04	0.057	1.1	0.0525	72	93
0.04	0.057	1	0.0525	74	92
0.04	0.055	1	0.0525	73	90
0.04	0.053	1	0.0525	74	92
0.04	0.052	1.1	0.0525	76	92
0.04	0.05	1	0.0525	77	92
0.04	0.05	1	0.0525	76	92
0.03	0.054	1.2	0.0525	74	88
0.04	0.06	1.4	0.0525	71	88
0.05	0.066	1.5	0.0525	69	88
0.05	0.059	1.1	0.04	63	85
0.05	0.055	3.8	0.08	55	73

## 4 Experimental Design, Materials and Methods

**Fig. 2:**
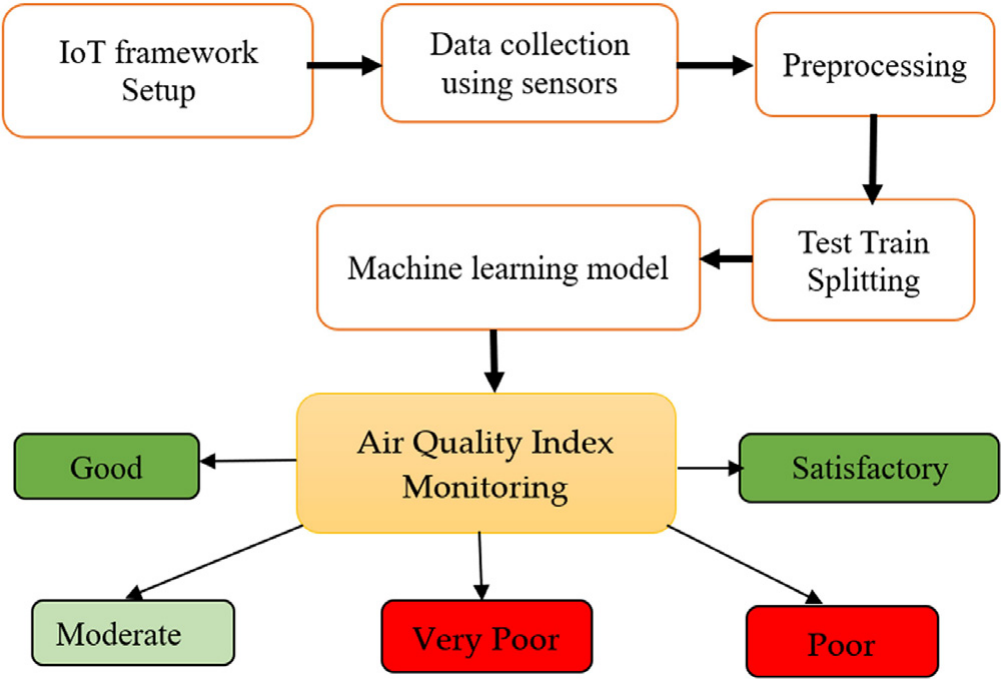
Workflow of designed method.

The proposed methodology is shown in Fig. 2. In this scenario, after getting the real-time data using the IoT framework, we applied machine learning techniques to monitor the AQI into Good, Satisfactory, Moderate, Poor and Very Poor.

**Fig.3:**
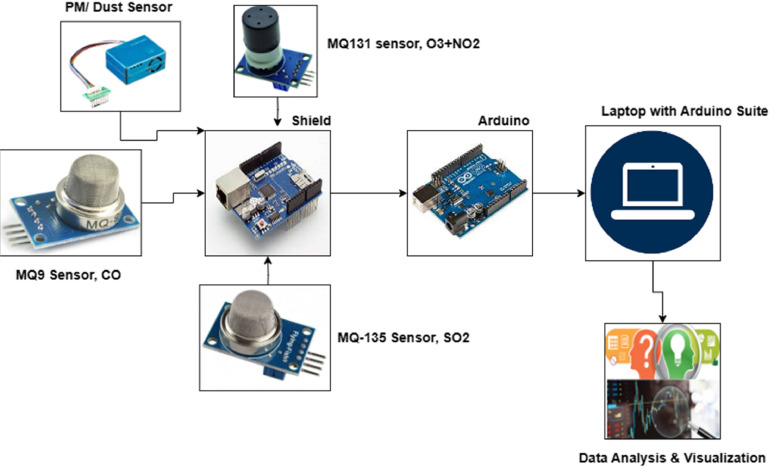
Block Diagram of proposed Methodology.

The physical part of the IoT framework is displayed in Fig. 3. MQ9 sensor is sued for CO, MQ131 is used for ozone and NO2, Dust sensor is used for PM 2.5 and PM 10 and MQ135 is used for SO2. Several researches on IoT based framework for real-time values have been done in this field like [[2], [3], [4]].

**Fig. 4:**
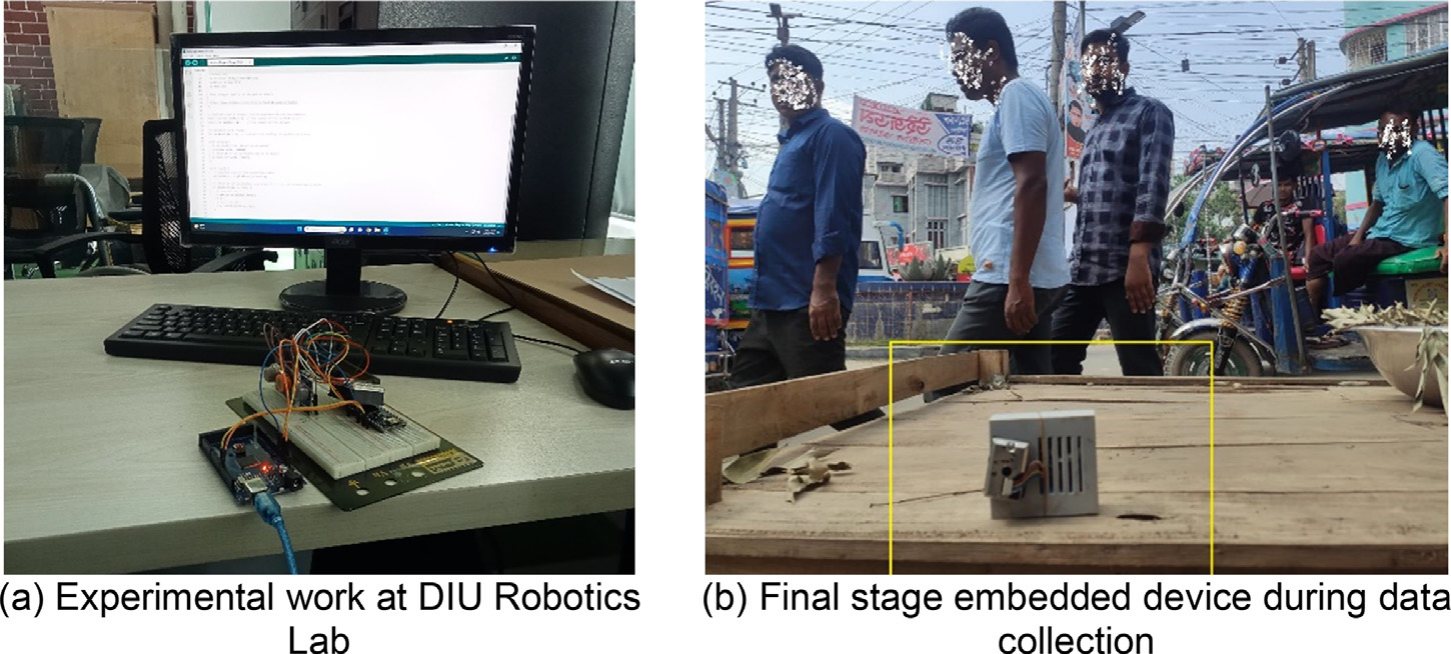
Some experimental images.

Fig. 4 shows some of the experimental images of the proposed system. The system design is done at Robotics Lab of the Department of Software Engineering in Daffodil International University shown in Fig. 4(a). The final embedded system during collecting real-time values is shown in Fig. 4(b).

### 4.1 Air quality index calculation

AQI index is calculated by Eq. (1).

(1) $$I_{i}=\left[\left(\frac{I_{max}-I_{min}}{B_{max}-B_{min}}\right)\times C_{p}-B_{min}\right]+I_{min}$$

where $B_{max}$ and $B_{min}$ are the breakpoints, $B_{max}$ means greater than or equal to given Concentration, and $B_{min}$ means smaller than or equal to given Concentration, $I_{max}=$ AQI value corresponding to $B_{max}$, $I_{min}=$ AQI value corresponding to $B_{min}$, $C_{p}=$ Pollutant concentration. Table 2 shows the breakpoints or range of AQI as for air quality and Table 3 describes the air pollution factors.

**Table 2:** AQI category range.

AQI Category	Range
Good	0–50
Satisfactory	51–100
Moderate	101–200
Poor	201–300
Very Poor	301–400

**Table 3:** Air pollution factors category range.

Category	PM2.5	NO2	O3	CO	SO2
Good	0–30	0–40	0–50	0–1.0	0–40
Satisfactory	31–60	41–80	51–100	1.1–2	41–80
Moderate	60–90	81–180	101–168	2.1–10	81–380
Poor	91–120	181–280	169–208	10.1–17	381–800
Very Poor	121–250	281–400	209–748	17.1–34	801–1600

Table 2, Table 3 show the AQI categories with the ranges of pollutants factors and pollutant concentration. Particle Matter 2.5 is measured in micrometers (µm), NO2, O3, and SO2 are all measured in Gram/cubic metre (g/m3), whereas CO is measured in Milligram per cubic meter (mg/m3). In addition, we calibrated before fieldwork in our Robotics Laboratory at Daffodil International University shown in Fig. 4(a). The results of the devices have been close to the standard values. We got the values 10 µm for PM 2.5, 40 g/m3 for NO2, 43 g/m3 for O3, 38 g/m3 for SO2 and 0 mg/m3 for CO.

### 4.2 Comparison performance metrics based on machine learning

**Fig. 5:**
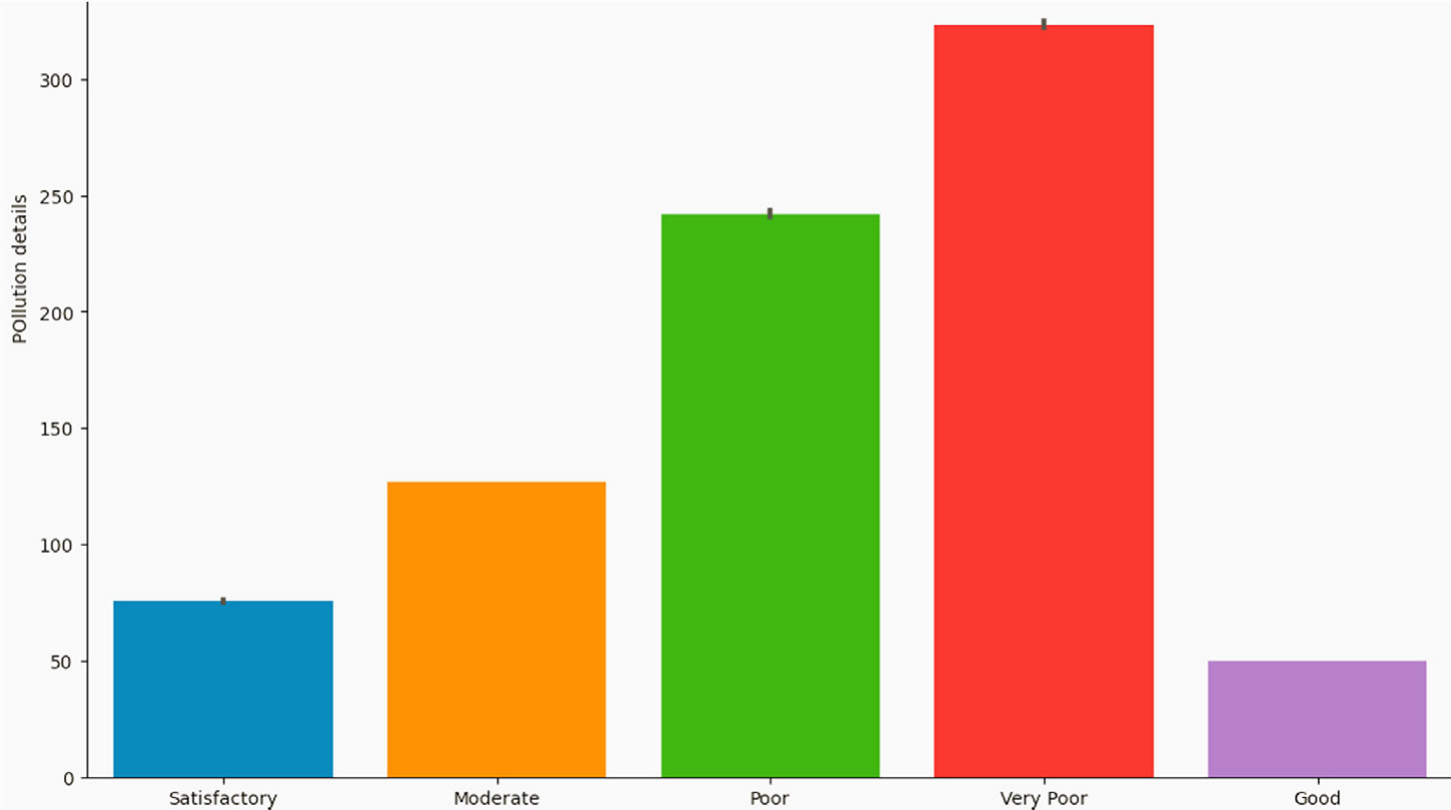
AQI values with the AQI Range.

We separate the dataset into two portion as dependent and independent before using ML models. In case of regression, we let AQI as the goal column, but in classification, AQI is as an independent column that targets AQI Range. Prior to using machine learning, the dataset is divided into training and test data. Fig. 5 displays the AQI values based on the AQI range. We evaluated the regression model using several matrices named root mean square error (RMSE), Mean Absolute Error (MAE), r2 score, and R-squared. The model is correct while the R-squared is high than RMSE. The accuracy, precision, recall, and F1 scores are all assessment matrices for the classification model.

In addition, we assessed the accuracy of the classifier model by calculating accuracy ratings for all classification models.

Table 4 shows the four models assessed in the ML Regressor Model: linear regression (LR), decision tree regression (DTR), random forest regression (RFR), and gradient boosting. Linear regression yields respectable results, with an MAE of 9.43 as well as an RMSE of 20.04. The model's R2 value is 0.505, which accounts for approximately 50.5 % of the variance. The Decision Tree Regressor shows more substantial errors, with an MAE of 11.76 along with an RMSE of 25.34. It has an astoundingly high R2 value of 0.995, suggesting a great fit to the data. The RFR produces better results, with an MAE of 9.00 and RMSE of 18.73. The model can explain over 93 % of the variance, according to the R2 value of 0.930. Gradient boosting is the strategy with the highest performance and the fewest errors (MAE of 8.34, RMSE of 17.86). The R2 value is 0.60, suggesting that the model can explain about 60 % of the variance. Gradient Boosting achieves the lowest errors and the highest R2 value, outperforming all other models. These findings highlight the effectiveness of gradient boosting in the regression issue.

**Table 4:** Regression score for ML model.

Model	MAE	RMSE	R Squared
LR	9.43	20.04	0.505
DTR	11.76	25.34	0.995
RFR	9.00	18.73	0.930
GB	8.34	17.86	0.60

Table 5 shows the assessment metric scores for several models, including precision, recall, F1 score, and accuracy. Random Forest Classifiers scored the highest across all parameters, suggesting good classification performance. The Random Forest model had a Precision score of 0.972 %, Recall score of 0.972 %, and F1 score of 0.972 %. The accuracy scores for Logistic Regression (LR) scoring are 93 %, DT scoring is 95 %, and K-NN scoring is 97.0 %. Naive Bayes scored 94 %, while the Random Forest Classifier (RFC) scored 97.2 %. The most accurate algorithm is RFC, which outperforms the others.

**Table 5:** Evaluation Metrics score table.

Model	Precision (%)	Recall (%)	F1 (%)	Accuracy (%)
LR	93.00	93.00	93.00	93.00
DTC	95.40	95.40	95.40	95.00
RFC	97.20	97.20	97.20	97.20
KNN	97.00	97.00	97.00	97.00
NB	94.00	94.00	94.00	94.00

These findings give valuable information about how well various algorithms perform, indicating that RFC may be especially well-suited to the classification problem. Researchers and practitioners may take these findings into account when selecting effective algorithms for analogous professions in the future.

## Limitations

From a highly populated area, the dataset was collected using IoT devices and it took almost five years. Due to the long-time data collection series, the system often experienced interruptions. However, for the data collection process, premium cloud storage was not used and the whole collection process was conducted in three places only.

## Ethics Statement

The authors have read and followed the ethical guidelines for publishing in Data in Brief, and they affirm that the present study does not involve human subjects, animal research, or data gathered from social media sites.

## CRediT authorship contribution statement

**Md. Monirul Islam:** Conceptualization, Methodology, Investigation, Formal analysis, Writing – original draft, Writing – review & editing. **Ferdaus Anam Jibon:** Methodology, Investigation, Formal analysis, Writing – review & editing. **M. Masud Tarek:** Conceptualization, Methodology, Investigation, Formal analysis, Writing – review & editing. **Muntasir Hasan Kanchan:** Conceptualization, Investigation, Formal analysis, Writing – review & editing. **Shalah Uddin Perbhez Shakil:** Conceptualization, Methodology, Investigation, Writing – original draft, Writing – review & editing.

## Data Availability

A Real-time Dataset of Air Pollution Monitoring Generated Using IoT (Original data) (Mendeley Data)
